# Clinical Utility of Urinary *β*2-Microglobulin in Detection of Early Nephropathy in African Diabetes Mellitus Patients

**DOI:** 10.1155/2017/4093171

**Published:** 2017-01-30

**Authors:** U. E. Ekrikpo, E. E. Effa, E. E. Akpan, A. S. Obot, S. Kadiri

**Affiliations:** ^1^University of Uyo and University of Uyo Teaching Hospital, Uyo, Nigeria; ^2^University of Calabar, Calabar, Nigeria; ^3^University College Hospital, Ibadan, Nigeria

## Abstract

*Background*. Studies have indicated that diabetic tubulopathy may occur earlier than glomerulopathy, therefore providing a potential avenue for earlier diagnosis of diabetic nephropathy. Urinary beta-2-microglobulin (*β*2m) was investigated in this study as a potential biomarker in the detection of early nephropathy in type 2 diabetics.* Methods*. One hundred and two diabetic subjects and 103 controls that met the inclusion criteria had data (sociodemographic, medical history, physical examination, and laboratory) collected. Urinary *β*2m levels and urinary albumin concentration (UAC) were determined.* Results*. Elevated urinary *β*2m was more frequent among the diabetics (52%, 95% CI: 42.1–61.8%) than among the controls (32%, 95% CI: 22.9–41.2%). The frequency of microalbuminuria was higher in the diabetics (35.3%, 95% CI: 25.9–44.7%) than in the controls (15.5%, 95% CI: 8.4–22.6%). There was a positive correlation between urinary *β*2m and UAC (rho = 0.38, *p* < 0.001). Multivariate analysis showed BMI (OR: 1.23, 95% CI: 1.05–1.45), eGFR (OR: 0.97, 95% CI: 0.94–0.99), and presence of microalbuminuria (OR: 3.94, 95% CI: 1.32–11.77) as independent predictors of elevated urinary beta-2-microglobulin among the diabetics.* Conclusion*. Urinary *β*2m may be useful, either as a single test or as a component of a panel of tests, in the early detection of diabetic nephropathy.

## 1. Introduction

Diabetic nephropathy is the single most common cause of end stage renal disease (ESRD) in adults worldwide [1–3]. In North America, diabetes mellitus accounts for 45% and 15% of incident dialysis patients in the USA and Canada, respectively [4, 5]. Diabetes mellitus comprised 51% of incident patients started on renal replacement therapy in Canada [5]. Data from European renal registries show a 12% increase in DM related ESRD with a primary diagnosis of DM in 20–44% of patients [2, 3]. In Japan, more than 40% of incident ESRD patients have diabetes mellitus [6]. In Africa, reports from Tunisia indicate an annual increase of 16% in the rate of diabetes mellitus as a cause of ESRD [7]. Congo's experience shows that about 39% of the diabetics in Kinshasa have chronic kidney disease coming second only to hypertension as a major risk factor for developing chronic kidney disease [8].

The chronic kidney disease picture in Nigeria is somewhat different. Hospital based studies in various parts of Nigeria have shown diabetes mellitus as the third commonest cause of end stage renal disease following chronic glomerulonephritis and hypertensive nephrosclerosis [9–11], but it may not remain so for long. With the ongoing epidemiologic transition [12] in sub-Saharan Africa, the prevalence of diabetes mellitus and other chronic noncommunicable diseases may be on the rise [13]. There are already reports of increased DM prevalence related to a constellation of risk factors including overweight individuals, family history of diabetes mellitus, and heavy alcohol consumption [14, 15] which in themselves are CKD risks.

Associated with the increasing prevalence of diabetes mellitus is a concomitant increase in the incidence of diabetic nephropathy [16]. We may soon see diabetic nephropathy as the commonest cause of end stage renal disease in Nigeria, resembling the scenario in the developed world. This has already occurred in other parts of Africa like Egypt where the prevalence of diabetic ESRD steadily increased from 8.9% in 1996 to 14.5% in 2001 with a coincident increased mortality risk [17].

Clinical evaluation of renal function in diabetics, for many years, had involved the use of serum creatinine and the various estimations of the glomerular filtration rate using creatinine based formulae. This has its accuracy limitations as it will only detect more advanced cases of diabetic nephropathy [18]. Other methods for the assessment of GFR are either too cumbersome or too expensive to be used in a clinical setting. Renal biopsy is not routinely done and the finding of classical Kimmelstiel-Wilson lesions and other structural lesions like glomerular basement membrane thickening from a biopsy specimen may actually indicate an advanced disease stage.

More recently, attention has been focused on the use of persistent microalbuminuria to define the presence of incipient diabetic nephropathy [19] and initial work on microalbuminuria attributed excretion of >30 mg/day of albumin in urine to increased glomerular filtration of albumin. Though the glomerular origin of microalbuminuria has not been contested, studies in rodents and man have shown that impaired tubular reabsorption of albumin at the proximal convoluted tubule is partly responsible for microalbuminuria [20, 21]. One study suggests that the initial renal damage resulting in microalbuminuria is the loss of charge-dependent tubular protein reabsorption occurring prior to the damage of the glomerular charge barrier in diabetics [22] while another has shown in diabetic children that tubular proteinuria actually predates microalbuminuria [23]. We have previously demonstrated early tubular dysfunction in African diabetics using urinary NAG suggesting that tubular dysfunction may precede glomerular damage [24].

The foregoing suggests that investigations targeting the tubular function in diabetics may be of immense clinical benefit in detecting early diabetic nephropathy, possibly earlier than the occurrence of persistent microalbuminuria. This study investigates the clinical utility of urinary beta-2-microglobulin levels in detecting early nephropathy in African diabetics.

## 2. Methods

This was a single centre, observational cross-sectional study involving patients with diabetes mellitus seen in the medical outpatient clinic of the University of Uyo Teaching Hospital, Uyo. One hundred and thirteen diabetic patients who met the inclusion criteria and did not have any of the exclusion criteria described below were recruited using simple random sampling into the study. Exclusion criteria included individuals with end stage kidney disease [25], a history of urinary tract infection in the one month preceding the interview, renal ultrasound features suggestive of structural urinary tract abnormalities [26], diseases associated with increased serum beta-2-microglobulin (HIV disease [27], lymphomas, multiple myeloma, and connective tissue disease [28]), and history of aminoglycoside use in the two weeks preceding the day of the interview [26]. A group of 113 nondiabetic individuals who did not have any of the conditions listed in the exclusion criteria were also recruited. Height, weight, waist and hip circumference, and blood pressure were measured using standard techniques.

The urinary *β*2-microglobulin (*β*2m) level in the participants was assayed using the beta-2-microglobulin ELISA kit, EIA 3609, from DRG Diagnostics International Inc., USA, which has precision of 0.1 *μ*g/mL. The average coefficient of variation for the plate control means was 10.4%. The proportion of participants with a urinary *β*2-microglobulin level >0.3 *μ*g/mL (upper limit of normal for urinary *β*2m from ELISA kit information) was adjudged to have elevated urinary *β*2-microglobulin level. The presence of albuminuria was measured using MICROALBUMIN™, an immunoturbidimetric assay from Fortress Diagnostics Limited, Antrim Technology Park, United Kingdom. The patients were to have 3 urine samples collected monthly over a 3-month period. Those with at least 2 out of the 3 samples were deemed to have persistent microalbuminuria. Individuals who did not have all their samples collected were excluded from the analysis.

Creatinine estimation was determined using Jaffe's alkaline picrate kinetic method. The glomerular filtration rate was computed using the Cockroft-Gault, 4-variable MDRD and CKD-EPI formulae [29–31]. HbA1c levels were determined using the glycohemoglobin reagent colorimetric set from TECO Diagnostics, Anaheim, California 92807, USA. Total cholesterol, triglycerides, and HDL were assayed using RANDOX kits while LDL was derived from the Friedewald equation [32].

Data analysis was performed using STATA 10 (StataCorp, College Station, TX, USA). The baseline sociodemographic and clinical characteristics of the participants were analyzed. Mean ± standard deviation was computed for normally distributed continuous variables and median and their corresponding interquartile range for continuous variables that are not normally distributed. Pearson's Chi-square test (or Fisher's exact test) was used to compare categorical variables while Student's *t*-test (or its nonparametric equivalent) was employed in comparing quantitative variables. Univariate logistic regression was used to identify factors associated with elevated urinary *β*2-microglobulin. Factors with a *p* value of the Wald statistic <0.25 and those found in the literature review to be risk factors for diabetic nephropathy were included in the multivariate logistic regression. The assumption of linearity in the model was assessed by including polynomial functions of the continuous variables into the final model. A Receiver Operator Characteristic (ROC) curve for the model was used to assess the fit of the model. Ethical clearance was obtained from the University of Uyo Teaching Hospital Human Research Ethics Committee.

## 3. Results

One hundred and thirteen subjects and 113 controls were recruited into the study. Twenty-one [11 (9.7%) of the subjects and 10 (8.8%) controls] were lost to follow-up. Data from 102 subjects and 103 controls was available for analysis after excluding individuals who were lost to follow-up. Tables 1 and 2 show the sociodemographic, clinical, and laboratory characteristics of the study participants.

The subjects (diabetics) had a significantly higher proportion of individuals with tertiary and secondary levels of education compared to the controls. The age and gender distributions were similar for both groups and the proportion of married individuals in both groups was similar. The subjects had a significantly higher proportion of individuals with a family history of diabetes mellitus (42.2% versus 10.7%, *p* < 0.001). There was no significant difference in the proportion of individuals in both groups who had a positive family history of hypertension (25.2% for the subjects versus 26.5% for the controls, *p* = 0.94) but a family history of kidney disease was more prevalent among the subjects (7.8% compared to 2.9%) than the controls.

The hemoglobin levels and high-density lipoprotein were similar for both groups. The other laboratory parameters were found to be significantly different. The subjects had higher serum creatinine, urea, uric acid, glycated hemoglobin, and random plasma glucose compared to the control group. The LDL-cholesterol, total cholesterol, triglycerides, and atherogenic ratio were significantly higher in the subjects than in the controls. Among the subjects, 80.4% had at least one fraction of the lipid profile being deranged. This proportion increased to 86.8% among the diabetics with elevated urinary beta-2-microglobulin. This consisted of high LDL-cholesterol in 55.9%, high triglycerides in 70.6%, high total cholesterol in 40.2%, and low HDL-cholesterol in 48.0%. There were 65 (63.7%) subjects with poor long-term glycemic control defined as glycated hemoglobin greater than 6.5%.

## 4. Frequency of Elevated Urinary ***β***2m

The subjects had median urinary *β*2m of 0.41 *μ*g/mL (interquartile range: 0.1–0.99 *μ*g/mL) while the controls had median urinary *β*2m of 0.1 *μ*g/mL (IQR: 0.1–0.41). This difference was statistically significant. The proportion of subjects who had elevated urinary *β*2m was 52.0% (95% CI: 42.1–61.8%) compared to the 32.0% (95% CI: 22.9–41.2%) of the controls, *p* = 0.004. There was no gender difference in elevated urinary *β*2m among the subjects [50.0% (19) of males versus 53.1% of the female diabetics, *p* = 0.76]. There were 19 (43.2%) male controls with elevated urinary *β*2m compared to 14 (23.7%) female controls, *p* = 0.03.

There was no difference in the proportion of subjects with elevated urinary *β*2m between those with DM and hypertension and those with DM only. Fourteen (46.7%) of the 30 in the “DM only” group had elevated urinary *β*2m compared to 39 (54.2%) of the 72 with diabetes and hypertension, *p* = 0.49. Finally, among the controls, of the 49 with hypertension, 20 (40.8%) had elevated urinary *β*2m compared to 13 (24.1%) of the 54 controls without hypertension, *p* = 0.07.

Table 3 shows the correlations and *p* values for the associations with *β*2m and UAC, systolic and diastolic blood pressure, estimated GFR, the components of the lipid profile, and serum uric acid.

## 5. Predictors of Elevated ***β***2m among the Diabetics

Univariate and multivariate regression models (Table 4) were used to investigate factors that independently predict the occurrence of elevated *β*2m in the diabetic population. At the univariate level, diabetics with microalbuminuria were 5 times more likely to have elevated *β*2m. Other significant associations with elevated urinary *β*2m at the univariate level included BMI (6% increased risk), eGFR (2% reduced risk), LDL (46% increased risk), triglycerides (93% increased risk), and increased atherogenic ratio (20% increased risk).

At the multivariate level, diabetics with persistent microalbuminuria had a nearly 4-fold increased risk of having tubular dysfunction as measured by elevation in urinary *β*2m excretion after adjusting for the effects of changes in eGFR, presence of hypertension, age, gender differences, dyslipidemia, BMI, waist circumference, HbA1c levels, and duration of diabetes mellitus.

## 6. Comparison of the Utility of Urinary ***β***2m and Microalbuminuria in Detecting Early Diabetic Nephropathy

Table 5 shows comparable correlation parameters between albumin creatinine ratio and eGFR and between urinary *β*2m and eGFR among the subjects.

Among the normoalbuminuric subjects, 25 (37.9%) already had elevated urinary *β*2m detected while elevated urinary *β*2m was seen in 27.6% of the normoalbuminuric controls. This difference was statistically significant, *p* < 0.001.

Among the subjects with normal urinary *β*2m, only 8 (16.3%) had microalbuminuria. Only 7 (10.0%) of the controls with normal urinary *β*2m had microalbuminuria. The comparison of urinary *β*2m and microalbuminuria in the detection of early DN is shown in Table 6.

There was a significantly greater proportion of normoalbuminuric diabetics with elevated urinary *β*2m than normal urinary *β*2m with microalbuminuria.

## 7. Discussion

This study examines the clinical utility of urinary *β*2m excretion, a measure of renal tubular dysfunction, in the early diagnosis of diabetic nephropathy.

The median level of urinary *β*2m as well as the proportion of diabetics with elevated urinary *β*2m was significantly higher in the diabetics than in controls. Indeed, over half of the diabetics had elevated urinary *β*2m suggesting the presence of tubulopathy. Increased *β*2m levels in diabetics have been reported by other studies [33, 34] just as other studies have found proportions of diabetic tubulopathy in type 2 diabetes in the range of 55 to 57% using urinary *β*2m as a measure of tubulopathy [35]. We observed a linear relationship between urinary *β*2m and serum creatinine as well as a significant inverse relationship between GFR and urinary *β*2m in this diabetic population. These findings suggest that urinary *β*2m mirrors reasonably well increases in serum creatinine as renal function declines. This has been demonstrated elsewhere [36]. Apakkan Aksun et al. documented a significant positive correlation between urinary *β*2m and serum creatinine and a negative correlation with GFR estimated from the uptake phase of 99m technetium diethylenetriaminepentacetate renogram (GFR-DTPA) and creatinine clearance [34]. As serum creatinine increases and eGFR reduces, there is a concomitant worsening of diabetic tubulopathy manifesting itself as a rise in urinary *β*2m. Since this is a cross-sectional study, it is difficult to conclude whether the glomerulopathy leading to rising serum creatinine and reducing GFR occurred earlier than the tubulopathy or whether the tubulopathy led to the glomerular dysfunction. It is also likely that both lesions occurred concurrently. Both questions would best be addressed by a cohort study.

Multivariate analysis showed BMI as a predictor of elevated urinary *β*2m levels leading to a 23% increased risk of tubular dysfunction for every one unit increase in BMI among the diabetics after adjusting for all the other factors in the model. There have been arguments for and against the role of BMI in the progression of diabetic nephropathy. A retrospective cohort study refuted the idea of a significant effect of BMI on diabetic nephropathy after a 3-year follow-up period of type 2 diabetics [37] when analysis showed no association between eGFR and BMI. Many other workers believe obesity encourages progression of nephropathy of most aetiologies because of its strong association with albuminuria and nephrosclerosis. Waist circumference, a measure of abdominal obesity, did not have a significant influence on the development of diabetic tubulopathy in this study. It is therefore difficult to conclude from this study that higher BMI can encourage progression of diabetic nephropathy. Larger longitudinal studies on the effect of BMI on diabetic nephropathy among Blacks may help answer this question as this was not the primary aim of this study.

The other significant factors predisposing to high urinary *β*2m included eGFR and microalbuminuria. After adjusting for the effect of age, gender, duration since diagnosis of diabetes mellitus, lipid profile, BMI, waist circumference, hypertension, and glycated hemoglobin levels, there was a 3% reduction in the risk of developing elevated urinary *β*2m for every one unit increase in the eGFR. This suggests that as renal function worsens (declining eGFR), the amount of urinary *β*2m increases. This inverse relationship was also observed at the univariate analysis level of this study. This strong relationship between GFR and microalbuminuria has led to a suggestion that urinary *β*2m could be used either alone or as part of a panel of tests in the early diagnosis of diabetic nephropathy [34].

The subjects with microalbuminuria had a demonstrably significant fourfold increased likelihood of also having elevated urinary *β*2m compared to those with normoalbuminuria after adjusting for all the other factors in the multivariate model. The rising levels of urinary albumin concentration as urinary *β*2m increases may not be solely because of simultaneous tubular and glomerular dysfunction in diabetics but may possibly also be due to an earlier tubular dysfunction resulting in impaired reabsorption at the proximal tubules of albumin filtered through normal glomeruli [38]. This is one of the reasons why some consider tubular dysfunction to possibly occur earlier than glomerular dysfunction in diabetics.

More than a third of the normoalbuminuric diabetics in this study had already developed elevated levels of urinary *β*2m. On the other hand, only 16.3% of diabetics with normal urinary *β*2m had microalbuminuria. This difference in proportions was statistically significant suggesting that increases in urinary *β*2m may occur earlier than microalbuminuria. This finding appears to be in support of the newer pathogenetic theories of diabetic nephropathy that suggest that diabetic tubulopathy occurs earlier than diabetic glomerulopathy [38]. A study had documented up to 55% of normoalbuminuric diabetics with elevated urinary *β*2m [35]. Several explanations have been put forward including a strong correlation between glomerular hyperfiltration (one of the earliest features of glomerular dysfunction) in normoalbuminuric patients and fractional reabsorption of sodium at the proximal tubules [39]; secondly, the finding of a higher risk of progression to microalbuminuria from normoalbuminuria in individuals with increased kidney volume which is mainly due to tubular hypertrophy and interstitial expansion; and thirdly, the occurrence of increased proximal tubule reabsorption leading to glomerular hyperfiltration as a result of glomerulotubular feedback. Glomerulotubular feedback may explain the reduction in GFR after an initial rise in early diabetic nephropathy whereas tubulointerstitial damage (especially atrophic proximal tubules) leads to reduced reabsorption of sodium and therefore reduced GFR.

Another factor to consider in the relationship between *β*2m and microalbuminuria is the relatively smaller molecular weight of *β*2m compared to albumin (one-sixth of albumin's molecular weight). This property encourages free filtration of *β*2m across the nondamaged filtration barrier. Smaller quantities of albumin are filtered across the “normal” filtration barrier and may require glomerular damage for larger quantities to be seen in urine. In the setting of tubulopathy, reabsorption of *β*2m is impaired and urinary *β*2m becomes elevated early in the course of diabetes mellitus. Moreover, for microalbuminuria to occur, the tubular mechanisms for reabsorption of proteins must have been overwhelmed by large quantities of albumin presented to the proximal tubules as a result of glomerulopathy [40]. This means microalbuminuria is not entirely a result of glomerular dysfunction but also has a tubular component to it.

It may be difficult from the findings of this study to conclude that elevated urinary *β*2m occurs earlier than microalbuminuria but one can safely say that elevated urinary *β*2m occurs at least as early as microalbuminuria in the course of diabetic nephropathy and there is a suggestion that urinary *β*2m elevation may occur earlier. This has led to the proposal to use a panel of urinary markers (including beta-2-microglobulin) to increase the chances of detecting early nephropathy in diabetics.

## Figures and Tables

**(a):** 

	Subjects (*n* = 102)	Controls (*n* = 103)	*p*
Age (years, mean ± SD)	54.8 ± 10.1	53.2 ± 12.0	0.30^*∗*^
Female gender, *n* (%)	64 (62.8)	59 (58.1)	0.43^†^
Married individuals, *n* (%)	90 (90.9)	83 (93.3)	0.69^†^
Educational status, *n* (%)			
No formal education	8 (8.2)	18 (20.2)	0.02^†^
Primary	19 (19.4)	22 (24.7)	
Secondary	11 (11.2)	3 (3.4)	
Tertiary	60 (61.2)	46 (51.7)	

^*∗*^Student's *t*-test. ^†^Chi-square test.

**(b):** 

	Subjects (*n* = 102)	Controls (*n* = 103)	*p*
Family history of diabetes mellitus	43 (42.2%)	11 (10.7%)	<0.001
Family history of hypertension	27 (26.5%)	26 (25.2%)	0.94
Family history of kidney disease	8 (7.8%)	3 (2.9%)	<0.001
History of frothy urine	46 (45.1%)	5 (4.9%)	<0.001
History of hematuria	7 (6.9%)	1 (0.9%)	0.03^†^
Body mass index (kg/m^2^)^*∗∗*^	26.9 ± 4.5	26.2 ± 6.1	0.34^Δ^
Waist circumference (cm)^*∗∗*^	95.8 ± 12.6	89.0 ± 15.3	0.007^Δ^
Hip circumference (cm)^*∗∗*^	99.6 ± 13.6	96.2 ± 14.6	0.09
Waist-to-hip ratio^$^	0.94 (0.88–1.01)	0.93 (0.88–0.99)	0.17^*∗*^
Systolic blood pressure (mmHg)^*∗∗*^	134.2 ± 22.4	139.3 ± 22.1	0.10
Diastolic blood pressure (mmHg)^*∗∗*^	82.3 ± 11.5	80.4 ± 13.7	0.29
mABP (mmHg)^$^	100.5 (93.3–108.3)	97.3 (86.3–109.3)	0.15

^†^Fisher's exact test. ^*∗*^Wilcoxon rank sum test. ^Δ^Student's *t*-test. ^$^Median (IQR). ^*∗∗*^Mean (SD). mABP: mean arterial blood pressure.

**Table 2:** Comparison of laboratory findings in both groups.

	Diabetics (*n* = 102)	Controls (*n* = 103)	*p*
Serum creatinine (*μ*mol/L)^$^	91 (74–102)	84 (69–98)	0.03
Serum urea (mmol/L)^$^	4.6 (3.5–5.1)	3.7 (2.6–4.9)	0.003
Hemoglobin (g/dL)^$^	13.8 (13–14.8)	14.2 (13.6–14.8)	0.14
Serum uric acid (mmol/L)^*∗∗*^	5.6 ± 1.5	5.0 ± 1.5	0.01
Random plasma glucose (mmol/L)^*∗∗*^	12.0 ± 3.9	6.0 ± 1.6	<0.001
Glycated hemoglobin (%)^$^	7.1 (5.3–8.1)	4.6 (3.9–5.2)	<0.001
Total cholesterol (mg/dL)^*∗∗*^	5.0 ± 1.4	3.5 ± 1.3	<0.001
Triglycerides (mg/dL)^*∗∗*^	2.13 ± 0.93	1.40 ± 0.81	<0.001
LDL-cholesterol (mg/dL)^$^	3.0 (1.4–3.9)	1.4 (1.02–1.7)	<0.001
HDL-cholesterol (mg/dL)^$^	1.1 (0.86–1.2)	0.98 (0.86–1.2)	0.83
Atherogenic ratio (total cholesterol/HDL)^$^	4.4 (3.2–6.9)	3.3 (2.4–4.6)	<0.001
UAC (mg/L)^$^	23 (10–100)	0.36 (0.3–10.2)	<0.001
Estimated GFR_CG_ (mL/min)^$^	68 (54–87)	81 (58–104)	0.02
Estimated GFR_MDRD_ (mL/min/1.73 m^2^)^$^	82.7 (67.1–97.9)	88.7 (76.2–117.8)	0.01
Estimated GFR_CKD-EPI_ (mL/min/1.73 m^2^)^$^	82.5 (67.4–97.3)	90.0 (75.7–109.8)	0.004
Urinary *β*2m (*μ*g/mL)^$^	0.41 (0.1–0.99)	0.1 (0.1–0.41)	0.002

UAC: urinary albumin concentration; *β*2m: beta-2-microglobulin. ^*∗∗*^Mean (SD). ^$^Median (IQR).

**Table 3:** Correlation of *β*2m with selected parameters in subjects and controls.

	Total (*N* = 205)		Subjects (*n* = 102)		Controls (*n* = 103)	
	Rho	*p*	Rho	*p*	Rho	*p*
Age	0.04	0.59	0.08	0.41	−0.03	0.80
Urinary albumin concentration	0.38	<0.001	0.34	0.004	0.28	0.004
eGFR_(CG)_	−0.19	0.01	−0.31	0.002	−0.02	0.84
Serum creatinine	0.43	<0.001	0.45	<0.001	0.37	0.001
Serum urea	0.14	0.04	0.13	0.18	0.11	0.28
Serum uric acid	0.16	0.02	0.17	0.08	0.04	0.71
Total cholesterol	0.32	<0.001	0.33	0.001	0.14	0.15
Triglycerides	0.21	0.002	0.22	0.02	0.007	0.94
LDL-cholesterol	0.30	0.002	0.21	0.17	−0.06	0.64
HDL-cholesterol	−0.03	0.71	−0.12	0.21	0.04	0.71
Glycated hemoglobin	0.22	0.002	0.15	0.12	0.06	0.55

**Table 4:** Univariate and multivariate logistic regression models for factors predicting elevated *β*2m.

	Univariate		Multivariate^*∗*^	
	Odds ratio (95% CI)	*p* value	Odds ratio (95% CI)	*p* value
Age (years)	1.01 (0.97–1.04)	0.75	0.99 (0.95–1.05)	0.89
Male gender	0.84 (0.38–1.86)	0.67	1.17 (0.36–3.79)	0.80
Positive hypertension status	1.3 (0.56–2.99)	0.54	0.60 (0.20–1.80)	0.36
Duration of DM	1.00 (0.99–1.01)	0.82	0.99 (0.98–1.03)	0.17
Waist circumference	1.00 (0.97–1.03)	0.97	0.98 (0.93–1.03)	0.39
BMI	1.06 (0.97–1.16)	0.19	1.23 (1.05–1.45)	0.01^*∗∗*^
Positive microalbuminuria	5.79 (2.30–14.59)	<0.001	3.94 (1.32–11.77)	0.01^*∗∗*^
eGFR	0.98 (0.97–0.99)	0.04	0.97 (0.94–0.99)	0.04^*∗∗*^
LDL-C	1.46 (1.08–1.99)	0.02	1.32 (0.71–2.43)	0.38
Total cholesterol/HDL	1.20 (1.03–1.41)	0.02	0.93 (0.71–1.24)	0.64
Triglycerides	1.93 (1.16–3.22)	0.01	1.85 (0.96–3.54)	0.06
HbA1c	1.18 (0.98–1.42)	0.07	1.09 (0.86–1.38)	0.47

^*∗*^The area under the Receiver Operator Characteristic (ROC) curve of the model was 0.80. ^*∗∗*^Statistically significant after adjusting for other variables.

**Table 5:** Comparison of correlation of *β*2m with eGFR versus UAC with eGFR in subjects and controls.

	Subjects (*n* = 102)		Controls (*n* = 103)	
	Rho	*p*	Rho	*p*
UAC	−0.29	0.01	−0.19	0.06
*β*2m	−0.31	0.01	−0.02	0.84

**Table 6:** Comparison of urinary *β*2m and microalbuminuria in detecting early DN.

	Microalbuminuria	
	Positive	Negative
Elevated urinary *β*2m		
Positive	28 (77.8%)	25 (37.9%)
Negative	8 (16.3%)	41 (62.1%)

McNemar *X* = 8.76, *p* = 0.003.

## Additional Points

*Limitation*. The cross-sectional design of this study has limited our ability to document the temporal profile of the studied biomarkers in the evolution of diabetic nephropathy. A prospective cohort study of initially normoalbuminuric diabetic patients would have answered this question better.
